# Role of Low-Molecular-Mass Penicillin-Binding Proteins, NagZ and AmpR in AmpC β-lactamase Regulation of *Yersinia enterocolitica*

**DOI:** 10.3389/fcimb.2017.00425

**Published:** 2017-09-27

**Authors:** Chang Liu, Chuchu Li, Yuhuang Chen, Huijing Hao, Junrong Liang, Ran Duan, Zhaoke Guo, Jing Zhang, Zhongzhi Zhao, Huaiqi Jing, Xin Wang, Shihe Shao

**Affiliations:** ^1^Department of Pathogenic Biology, School of Medical Science, Jiangsu University, Zhenjiang, China; ^2^National Institute for Communicable Disease Control and Prevention, Chinese Center for Disease Control and Prevention, State Key Laboratory for Infectious Disease Prevention and Control, Collaborative Innovation Center for Diagnosis and Treatment of Infectious Diseases, Beijing, China; ^3^Qinghai Institute for Endemic Diseases Prevention and Control, Xining, China

**Keywords:** *Yersinia enteocolitica*, AmpC β-lactamase, AmpD, PBPs, NagZ, AmpR

## Abstract

*Yersinia enterocolitica* encodes a chromosomal AmpC β-lactamase under the regulation of the classical *ampR-ampC* system. To obtain a further understanding to the role of low-molecular-mass penicillin-binding proteins (LMM PBPs) including PBP4, PBP5, PBP6, and PBP7, as well as NagZ and AmpR in *ampC* regulation of *Y. enterocolitica*, series of single/multiple mutant strains were systematically constructed and the *ampC* expression levels were determined by *luxCDABE* reporter system, reverse transcription-PCR (RT-PCR) and β-lactamase activity test. Sequential deletion of PBP5 and other LMM PBPs result in a continuously growing of *ampC* expression level, the β-lactamse activity of quadruple deletion strain YEΔ4Δ5Δ6Δ7 (*pbp4, pbp5, pbp6*, and *pbp7* inactivated) is approached to the YEΔD123 (*ampD1, ampD2*, and *ampD3* inactivated). Deletion of *nagZ* gene caused two completely different results in YEΔD123 and YEΔ4Δ5Δ6Δ7, NagZ is indispensable for YEΔ4Δ5Δ6Δ7 *ampC* derepression phenotype but dispensable for YEΔD123. AmpR is essential for *ampC* hyperproduction in these two types of strains, inactivation of AmpR notable reduced the *ampC* expression level in both YEΔD123 and YEΔ4Δ5Δ6Δ7.

## Introduction

*Yersinia enterocolitica*, a member of *Enterobacteriaceae*, is a zoonotic pathogen widely distributed in nature (Wang et al., 2011; Liang et al., 2012). Most *Y. enterocolitica* exhibits intrinsic resistance to β-lactm antibiotics by the production of chromosomally encoded β-lactamases called BlaA (a class A enzyme showing constitutive expression) and BlaB (an inducible AmpC-type β-lactamase), respectively (Cornelis and Abraham, 1975; Bent and Young, 2010).

The process of *ampC (blaB)* regulation is tightly linked to the peptidoglycan recycling and controlled by AmpG, AmpD, AmpR, and NagZ (Vollmer et al., 2008; Zeng and Lin, 2013). Briefly, peptidoglycan degradation products including GlcNAc-1,6-anhydromuropeptide is transported into the cytoplasm by AmpG and further hydrolyzedcosaminidase) to yielding 1,6-anhydromuropeptides, which is the AmpR activator ligand for *ampC* derepression (Zamorano et al., 2010; Huang et al., 2012; Yang et al., 2014). On the other hand, the stem peptides of GlcNAc-1,6-anhydromuropeptide and 1,6-anhydromuropeptides can be removed by AmpD (N-acetylmuramyl-l-alanine amidase) and eventually recycled into UDP-MurNAc-pentapeptide, which is the AmpR repressor ligand to repress *ampC* expression level (Juan et al., 2006; Balasubramanian et al., 2015; Liu et al., 2016). Penicillin-binding proteins (PBPs) also play an important role in *ampC* regulation (Sanders et al., 1997; Pfeifle et al., 2000). Recent studies have found that in *P. aeruginosa*, PBP4 (DacB), PBP5 (DacC), and PBP7 (PbpG) are involved in *ampC* regulation, and PBP4 is the major cause of *ampC* derepressed in clinical strains (Moya et al., 2009; Ropy et al., 2015).

Theoretically, NagZ is indispensable in chromosomal *ampC* derepression. In *P. aeruginosa, nagZ* inactivation dramatically reduces the β-lactam resistance of both PAOΔampD (*ampD* inactivation) and PAOΔ*dacB* (*pbp4* inactivation; Zamorano et al., 2010). However, although *nagZ* inactivation nearly abolished the basal-level derepressed β-lactamase activity of KJΔampDI (*ampD* inactivation), it did not affect the β-lactamase activity of KJΔmrcA (*pbp1a* inactivation) in *Stenotrophomonas maltophilia* (Huang et al., 2012).

Since the effects of the above-mentioned genes in *Y. enterocolitica* were seldom reported, we elucidated the role of low-molecular-mass penicillin-binding proteins (LMM PBPs) (PBP4, PBP5, PBP6, and PBP7), NagZ and AmpR in the *Y. enterocolitica* ampC regulation. Firstly, we investigated the effects of each LMM PBP on the expression of AmpC β-lactamase by monitoring the *ampC* promoter activity from a series of LMM PBPs mutant strains and confirmed by quantitative reverse transcription-PCR (qRT-PCR). Secondly, *nagZ* gene was deleted in two *ampC* derepressed strains YEΔD123 and YEΔ4Δ6Δ5Δ7 to determine the role for *ampC* expression.

## Materials and methods

### Bacterial strains, plasmids, primers, and growth conditions

Strains and plasmids used in this study were listed in Table 1. Individual genes were deleted initially from *Y. enterocolitica* subsp. palearctica 105.5R(r) (Wang et al., 2011). Luria-Bertani (LB) agar plates and broth were used as culture media for *Y. enterocolitica* (28°C) and *Escherichia coli* (37°C). For induction assay, cefoxitin was used according to the references (Guerin et al., 2015; Liu et al., 2016).

**Table 1:** Strains and plasmids used in this study.

**Strains or plasmid**	**Genotype or relevant characteristics**	**Source or references**
***Yersinia enterocolitica***		
105.5R(r)	Wild type; completely sequenced	Wang et al., 2011
YEΔZ	105.5R(r) *nagZ* deletion mutant	This work
YEΔD123	105.5R(r) *ampD1, ampD2, ampD3* triple mutant	Liu et al., 2016
YEΔD123ΔZ	105.5R(r) *ampD1, ampD2, ampD3, nagZ* quadruple mutant	This work
YEΔD123ΔR	105.5R(r) *ampD1, ampD2, ampD3, ampR* quadruple mutant	This work
YEΔ4	105.5R(r) *pbp4* deletion mutant	This work
YEΔ5	105.5R(r) *pbp5* deletion mutant	This work
YEΔ6	105.5R(r) *pbp6* deletion mutant	This work
YEΔ7	105.5R(r) *pbp7* deletion mutant	This work
YEΔ4Δ5	105.5R(r) *pbp4, pbp5* double mutant	This work
YEΔ4Δ6	105.5R(r) *pbp4, pbp6* double mutant	This work
YEΔ4Δ7	105.5R(r) *pbp4, pbp7* double mutant	This work
YEΔ5Δ6	105.5R(r) *pbp5, pbp6* double mutant	This work
YEΔ5Δ7	105.5R(r) *pbp5, pbp7* double mutant	This work
YEΔ6Δ7	105.5R(r) *pbp6, pbp7* double mutant	This work
YEΔ4Δ5Δ6	105.5R(r) *pbp4, pbp5, pbp6* triple mutant	This work
YEΔ4Δ5Δ7	105.5R(r) *pbp4, pbp5, pbp7* triple mutant	This work
YEΔ4Δ6Δ7	105.5R(r) *pbp4, pbp6, pbp7* triple mutant	This work
YEΔ5Δ6Δ7	105.5R(r) *pbp5, pbp6, pbp7* triple mutant	This work
YEΔ4Δ5Δ6Δ7	105.5R(r) *pbp4*, pbp5*, pbp6, pbp7*, quadruple mutant	This work
YEΔ4Δ5Δ6Δ7ΔZ	105.5R(r) *pbp4*, pbp5*, pbp6, pbp7, nagZ* quintuple mutant	This work
YEΔ4Δ5Δ6Δ7ΔR	105.5R(r) *pbp4*, pbp5*, pbp6, pbp7, ampR* quintuple mutant	This work
***E. coli***		
S17 λpir	λ-pir R6K(*thi thr leu ton lacY supE recA*::RP4-2Tc::Mu)	Simon et al., 1983
**PLASMIDS**		
pDS132	CmR; Conditionally replicating vector; R6K origin, mobRK4 transfer origin, sucrose-inducible *sacB*	Philippe et al., 2004
pΔNagZ	CmR; pDS132 containing 5′ and 3′ flanking sequence of *nagZ*	This work
pΔPBP4	CmR; pDS132 containing 5′ and 3′ flanking sequence of *pbp4*	This work
pΔPBP5	CmR; pDS132 containing 5′ and 3′ flanking sequence of *pbp5*	This work
pΔPBP6	CmR; pDS132 containing 5′ and 3′ flanking sequence of *pbp6*	This work
pΔPBP7	CmR; pDS132 containing 5′ and 3′ flanking sequence of *pbp7*	This work
pΔAmpR	CmR; pDS132 containing 5′ and 3′ flanking sequence of *ampR*	This work
pLUX*ampC*	CmR; pBBRlux containing promoter sequence of *ampC*	Liu et al., 2016
pNagZ	TcR; pSRKTc containing 105.5R(r) *nagZ* gene	This work

### Construction of *Y. enterocolitica* mutant strains

Knockout mutant strains were constructed using the method described previously (Chen et al., 2015; Liang et al., 2016; Liu et al., 2016). Briefly, the deletion mutants were constructed by double-crossover homologous recombination between wild-type strain chromosome and plasmids pΔNagZ, pΔAmpR, pΔPBP4, pΔPBP5, pΔPBP6l, and pΔPBP7. To evaluate the role of PBP4 (WP_005175403.1), PBP5 (WP_005158391.1) PBP6 (WP_023160783.1), and PBP7 (WP_005158897.1) in *Y. enterocolitica* 105.5R(r) *ampC* regulation, we constructed four single mutant strains: YEΔ4 (*pbp4* inactivation), YEΔ5 (*pbp5* inactivation), YEΔ6 (*pbp6* inactivation), and YEΔ7 (*pbp7* inactivation); six double mutant strains: YEΔ4Δ5, YEΔ4Δ6, YEΔ4Δ7, YEΔ5Δ6, YEΔ5Δ7, and YEΔ6Δ7; four triple mutant strains: YEΔ4Δ5Δ6, YEΔ4Δ5Δ7, YEΔ4Δ6Δ7, and YEΔ5Δ6Δ7; and one quadruple mutant strain: YEΔ4Δ5Δ6Δ7 (Table 1). The deletion mutants were identified by colony PCR firstly and then sequenced to confirm the in-frame deletion. Multiple deletion strains were sequentially constructed from the single mutant by use of the same procedure.

### Measurement of the *ampC* promoter activity

The method of measuring the *ampC* promoter activity with the *luxCDABE* reporter system was reported previously (Liu et al., 2016). The reporter plasmid pLUX*ampC* was transferred into the tested strains, and the luminescence was measured by using an Infinite M200 Pro spectrophotometer. The value of luminescence/OD600 was used to assess the *ampC* promoter activity.

### Determination of β-lactamase activity and antibiotic susceptibility testing

Specific β-lactamase activities were spectrophotometrically determined with nitrocefin (Oxoid) as a substrate as previously described (Liu et al., 2016). One unit of β-lactamase activity (U/mg) was defined as the number of nanomoles of nitrocefin hydrolyzed per minute per milligram of protein. Antibiotic susceptibility was determined using the standard 2-fold serial broth microdilution method according to the Guidelines of the Clinical Laboratory Standards Institute (CLSI, 2015).

### N-acetyl-β-glucosaminidase activity assay

The N-acetyl-glucosaminidase activity of the whole cell lysates of wild-type strain 105.5R(r) and YEΔZ were measured using 4-nitrophenyl N-acetyl-β-D-glucosaminide as a chromogenic substrate (Sigma). The presence of p-nitrophenol were detected by monitoring the optical density at 405 nm by 10 h continuously.

### Complementation assay

The ORF of *nagZ* was amplified and cloned into the broad-host-range expression vector pSRKTc to construct plasmid pNagZ. Transformants were selected on 10 μg/ml tetracycline *Yersinia* selective LB plates, acquisition of the appropriate plasmid was confirmed by colony PCR.

## Results

### Role of LMM PBPs in the expression of AmpC β-lactamase

**Figure 1:**
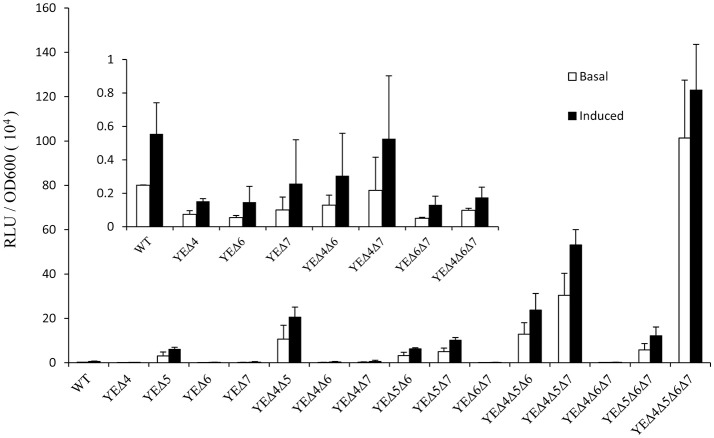
Analysis of the a*mpC* promoter activities in *Y. enterocolitica* 105.5R(r) wild-type strain and *pbp* mutants. The induction group was incubated with 40 μg/ml cefoxitin for 1 h. The error bars represent the standard deviations of triplicate tests.

After a series of LMM PBPs mutant strains were constructed, reporter plasmid pLUX*ampC* was used to monitor the *ampC* expression level (Liu et al., 2016). As shown in Figure 1, deletion *pbp5* caused a visible increase in the *ampC* promoter activity under both basal and induced conditions; but deletion of *pbp4, pbp6*, and *pbp7* did not affect the AmpC expression obviously. In the group of double and triple mutant strains, *ampC* derepression only appeared in Δ*pbp5* background, the *ampC* promoter activity of YEΔ4Δ5, YEΔ5Δ6, and YEΔ5Δ7 exhibited a marked rise compared with YEΔ4Δ6, YEΔ4Δ7, or YEΔ6Δ7. The level of *ampC* expression keep increasing in triple mutant strains YEΔ4Δ5Δ6, YEΔ4Δ5Δ7, and YEΔ5Δ6Δ7, but not in YEΔ4Δ6Δ7. Finally, the quadruple deletion strain YEΔ4Δ5Δ6Δ7 displayed the highest level of *ampC* promoter activity. These results suggested that PBP5 plays the most important roles in *Y. enterocolitica ampC* regulation. The qRT-PCR assay reconfirmed the results observed from *ampC* promoter activity assay (Table 2).

**Table 2:** Relative mRNA level of *ampC* in wild-type strain and its derived mutants.

**Strain**	**Relative mRNA level of *ampC*^a^**	
	**Basal**	**Induced^b^**
WT	1	1.3 ± 0.4
YEΔ4	1 ± 0.6	1.7 ± 0.5
YEΔ5	5.8 ± 3.5	7.8 ± 3.0
YEΔ6	1.2 ± 0.6	1.8 ± 0.6
YEΔ7	0.7 ± 0.4	1.2 ± 0.5
YEΔ4Δ5	10 ± 5	31 ± 16
YEΔ4Δ6	1 ± 0.2	1.4 ± 0.4
YEΔ4Δ7	0.7 ± 0.2	1.2 ± 0.5
YEΔ5Δ6	11 ± 1	15 ± 8
YEΔ5Δ7	7.7 ± 1.0	12 ± 4.8
YEΔ6Δ7	1.6 ± 0.3	2.4 ± 1.4
YEΔ4Δ5Δ6	22 ± 5	32 ± 18
YEΔ4Δ5Δ7	26 ± 4	41 ± 13
YEΔ4Δ6Δ7	2.1 ± 0.5	3.3 ± 1.6
YEΔ5Δ6Δ7	8.5 ± 1.0	12 ± 5
YEΔ4Δ5Δ6Δ7	42 ± 23	58 ± 10

a *Relative amount of mRNA compared to wild-type strain 105.5R(r) basal expression*.

b *Induction assay were performance with 40 μg/ml cefoxitin*.

### Role of NagZ in AmpC derepression of *Y. enterocolitica*

**Figure 2:**
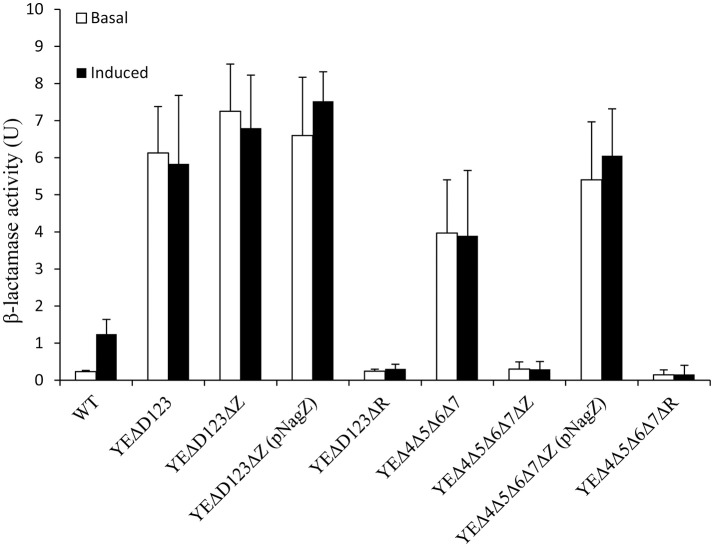
The role of AmpD, PBPs, NagZ, and AmpR in the β-lactamase expression of *Y. enterocolitica* by measuring the β-lactamase activity. These data are the average of three repeat experiments. The induction group was incubated with 40 μg/ml cefoxitin for 1 h. Error bars indicate the standard deviations of triplicate tests.

In agreement with our previous data (Liu et al., 2016), AmpD deletion strain YEΔD123 exhibit a derepression phenotype, and the β-lactamase activity of YEΔD123 is slightly higher than YEΔ4Δ5Δ6Δ7 (Figure 2). To evaluate the role of NagZ in AmpC derepression, *nagZ* gene was deleted in both derepression strains to construct YEΔD123ΔZ and YEΔ4Δ5Δ6Δ7ΔZ. As shown in Figure 2, *nagZ* was indispensable for *ampC* over expression of YEΔ4Δ5Δ6Δ7, the β-lactamase activity of *nagZ* deletion strain YEΔ4Δ5Δ6Δ7ΔZ was decreased significantly, closed to the wild-type strain level. In complementation assay, YEΔ4Δ5Δ6Δ7ΔZ (pNagZ) restored the β-lactamase activity to the level of YEΔ4Δ5Δ6Δ7. However, NagZ was dispensable in YEΔD123, the β-lactamase activity of *nagZ* deletion strain YEΔD123ΔZ was nearly as high as YEΔD123 (Figure 2). These results suggested that NagZ was needed in ΔPBPs-driven AmpC derepression, but did not perform its expected function in AmpD mutation strains. Antibiotic susceptibility test was also performed, as shown in Table 3, the MIC values of YEΔ4Δ5Δ6Δ7ΔZ were slightly below the wild-type strain 105.5R(r), far from its parent strain YEΔ4Δ5Δ6Δ7 for almost all tested β-lactams; but only a marginal distinction between YEΔD123 and YEΔD123ΔZ was found. These results illustrated that AmpD/PBPs regulate AmpC expression through NagZ dispensable/indispensable ways in *Y. enterocolitica*.

**Table 3:** The MIC values of β-lactam antibiotics in wild-type strain and its derived mutants.

**Antibiotic**	**MIC (mg/L) of antibiotic of strain^a^^,^^b^**				
	**WT**	**YEΔD123**	**YEΔD123ΔZ**	**YEΔ4Δ5Δ6Δ7**	**YEΔ4Δ5Δ6Δ7ΔZ**
**PENICILLINS**					
AMP	32	64	32	64	16
SAM	16	16	16	16	8
TIC	2	4	2	4	0.5
TZP	1	4	2	4	0.25
PIP	1	16	16	16	4
**CEPHALOSPORINS**					
CFZ	128	512	512	512	64
CAZ	0.25	2	1	2	0.5
FEP	0.25	0.25	0.125	0.06	0.03
CRO	≤0.125	0.5	0.25	0.5	0.125
**MONOBACTAM**					
ATM	≤0.125	0.5	0.5	1	0.12
**CARBAPENEMS**					
IPM	≤0.125	0.5	0.25	0.25	0.25
MEM	≤0.125	≤0.125	≤0.125	≤0.125	≤0.125
**LIPOPEPTIDES**					
CL	≤0.5	≤0.5	≤0.5	0.75	≤0.5

a *AMP, Ampicillin; SAM, Ampicillin-sulbactam; TIC, Ticarcillin; TZP, Piperacillin-tazobactam; PIP, Piperacillin; CFZ, Cefazolin; CAZ, Ceftazidime; FEP, Cefepime; CRO, Ceftriaxone; ATM, Aztreonam; IPM, Imipenem; MEM, Meropenem; CL, Colistin*.

b *MIC was determined in triplicate by standard two-fold serial broth microdilution method*.

### N-acetyl-β-glucosaminidase activity assay

**Figure 3:**
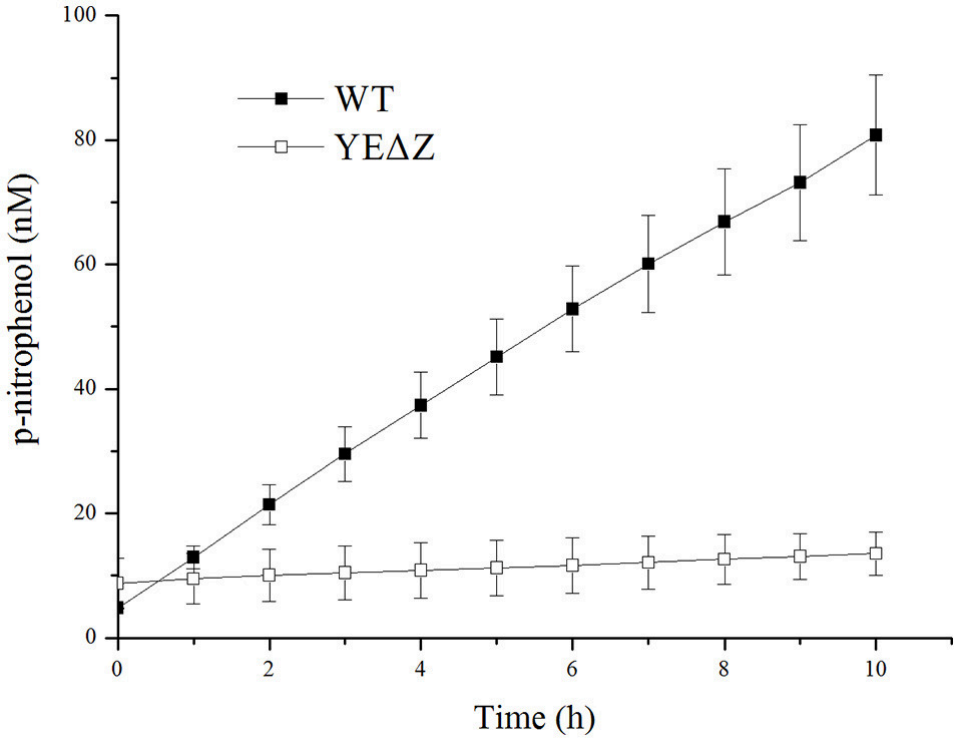
The N-acetyl-β-glucosamididase activity of wild-type *Y. enterocolitica* and *nagZ* deletion mutants was tested using 4-nitrophenyl N-acetyl-β-D-glucosaminide as a chromogenic substrate, and the p-nitrophenol present in the supernatant was measured at 405 nm.

The *nagZ* mutation strain YEΔZ was constructed, and determined by the enzyme activity of the both wild-type strain and YEΔZ for 10 h using N-acetyl-β-D-glucosaminide as substrate. As shown in Figure 3, YEΔZ abolished the N-acetyl-β-glucosaminidase activity completely, it was suggested that NagZ is the only enzyme that with N-acetyl-β-glucosaminidase activity in *Y. enterocolitica*.

### Role of AmpR in *ampC* expression of in *Y. enterocolitica*

In the paradigm of the *ampR-ampC* system, the *ampR* gene is located immediately adjacent to *ampC*, and AmpR plays a pivotal role in the regulation of AmpC (Seoane et al., 1992). To assess the role of AmpR in *Y. enterocolitica*, we compared the β-lactamase activity of YEΔD123ΔR, YEΔ4Δ5Δ6Δ7ΔR with their parent strains YEΔD123, YEΔ4Δ5Δ6Δ7, respectively. As a result, *ampR* inactivation dramatically reduced the β-lactamase activity of both YEΔD123ΔR and YEΔ4Δ5Δ6Δ7ΔR, regardless of adding cefoxitin or not (Figure 2).

## Discussion

The *ampR*-*ampC* system from *Citrobacter freundii* and *Enterobacter cloacae* has been well studied in the early 1990s (Lindberg et al., 1987; Peter et al., 1988). However, newly discovered *ampC* regulators such as, PBP4 (DacB) or NagZ in *Enterobacteriaceae* was not yet understood. A deep study in *Y. enterocolitica ampR-ampC* system would be helpful to improve the comprehensive understanding of *Enterobacteriaceae ampC* regulation.

PBPs are a group of enzymes involved in cell-wall recycling and the processes of AmpC β-lactamases regulation. In *E. coli* model, deletion of three or four PBPs and the concomitant inhibition of PBP 1a, 1b, and/or 2 results in an increased level of β-lactamase induction (Pfeifle et al., 2000). However, since *E. coli* lacks the chromosomal *ampR* gene, the result may be inconsistent with other members of the Gram-negative bacteria which have a chromosome encoding the *ampR-ampC* system. In 2009, Moya et al. demonstrated the inactivation of DacB (PBP4), a nonessential low-molecular mass PBPs is the principal reason for one-step high-level *ampC* expression in clinical strains of *P. aeruginosa* (Moya et al., 2009). Interestingly, inactivation of PBP4 in *E. cloacae* triggered a significant increase of β-lactams resistance, but without an obvious upregulation of *ampC* gene, it may be suggested that PBP4 regulates AmpC at a post-transcriptional level (Guerin et al., 2015). In this study, we found deletion of *pbp4* did not elevate the *ampC* expression level, this result is accordance with *E. cloacae*. After that, we deleted all four LMM PBPs one after another, and found that PBP5 is the most effective PBP involved in the regulation of *ampC* in *Y. enterocolitica*. Of the single-mutation strains, only the *pbp5* deletion strain YEΔ5 showed an obvious rise in *ampC* expression level. Likewise, for multi-mutation strains, the function of PBP4, PBP6, and PBP7 in *ampC* regulation were detected only if in Δ*pbp5* background. According to the results shown in Figure 1 and Table 2, we deduced the hierarchy of the role of PBPs genes in *ampC* derepression: PBP5 > PBP4 > PBP7 > PBP6. Although DacB may regulates AmpC at a post-transcriptional level (Guerin et al., 2015), but no trace of post-transcriptional mechanism has been found in *Y. enterocolitica*.

Along with the popular research of *ampC* regulation, there is growing evidence that some bacteria may regulate the expression of *ampC* through at least two different ways, one of which was NagZ-dependent, while the other worked without the participation of NagZ (Huang et al., 2012; Guerin et al., 2015). In the study on *P. aeruginosa, nagZ* inactivation was shown to attenuate *ampC* expression and was critical for basal-level *ampC* derepression in both PAΔD (*ampD* inactivation) and PAΔdB (*pbp4* inactivation) mutants (Asgarali et al., 2009; Zamorano et al., 2010). However, Δ*nagZ* had little effect on the cefoxitin-induced *ampC* expression level in both PAΔD and PAΔdB, which indicated that an unidentified non-NagZ product at work in this induction process. Furthermore, two different regulation ways of β-lactamase have been found in *S. maltophilia*, on one hand NagZ was essential for KJΔDI (*ampD* inactivation) *ampC* overexpression, on the other hand, *nagZ* inactivation hardly influenced the *ampC* expression level of KJΔmrcA (*pbp1a* inactivation; Huang et al., 2012). In this study, we also found two different *ampC* regulation ways exist in *Y. enterocolitica*, the patterns of which were just the reverse of that in *S. maltophilia* (Huang et al., 2012). The β-lactamase activity of YEΔD123 was not affected by the inactivation of the *nagZ* gene, whereas the introduction of Δ*nagZ* into the PBP mutation strain YEΔ4Δ5Δ6Δ7 dramatically reduced the β-lactamase activities at both the basal and induced level (Figure 2). As shown in Table 3, the antibiotic resistance of YEΔ4Δ5Δ6Δ7 and YEΔD123 were marked improved compare with wild-type strain, the MIC value of these two strains in TZP, PIP, CFZ, CAZ, CRO, and ATM is rising sharply. While after inactivation of *nagZ* gene simultaneously, only a marginal distinction between YEΔD123 and YEΔD123ΔZ was found, but the MIC values of YEΔ4Δ5Δ6Δ7ΔZ has shifted down significantly, far from its parent strain YEΔ4Δ5Δ6Δ7 for almost all tested β-lactams. To further confirm the function of NagZ, we constructed a *nagZ* deletion strain YEΔZ, and detected the N-acetyl-β-glucosaminidase activity of it to compare with the wild-type strain *Y. enterocolitica* 105.5R(r), the results showed that the ability of hydrolysis chromogenic substrate was completely lost in *nagZ* mutation strain YEΔZ (Figure 3), suggesting that NagZ (YE105_RS06670) was the only enzyme that possessed N-acetyl-β-glucosaminidase activity in *Y. enterocolitica* 105.5R(r). However, even though there is no readable N-acetyl-β-glucosaminidase activity in YEΔZ, we also did the bioinformatic search to look for possible NagZ homologs in genome to find the protein worked in YEΔD123ΔZ. According to the gene function annotation of 105.5R(r), we considered the YE105_RS13000 may have similar function with NagZ, but it was not clear if this protein participated the *ampC* regulation or not. Therefore, further studies needed to performed to elucidate the function of YE105_RS13000 in *Y. enterocolitica ampC* regulation.

In *Y. enterocolitica*, the function of AmpR was roughly the same as other members of *Enterobacteriaceae* or *P. aeruginosa*. The introduction of Δ*ampR* into the AmpC hyperproduction strains YEΔD123 and YEΔ4Δ5Δ6Δ7 resulted in a sharp decline in the *ampC* expression (Figure 2). The inducibility of YEΔD123ΔR and YEΔ4Δ5Δ6Δ7ΔR also disappeared completely (Lindberg et al., 1985; Lindberg and Normark, 1987).

In conclusion, in terms of AmpC β-lactamase regulation, *Y. enterocolitica* shared some common characteristics with *P. aerugiosa* and other members of *Enterobacteriaceae*, but it also had its own features. This was the first investigation to the characterization of *Y. enterocolitica ampC* regulation. It provided a more comprehensive understanding of the AmpC β-lactamase regulation in Gram-negative bacteria.

## Author contributions

CL, CCL, SS, HJ, and XW designed the experiment together. YC and HH performed data analysis. JL and RD participated in the manuscript translation. ZG, JZ, and ZZ contributed to finish the work. All authors contributed to writing of the manuscript.

### Conflict of interest statement

The authors declare that the research was conducted in the absence of any commercial or financial relationships that could be construed as a potential conflict of interest.
